# Evaluation of the role of *STAP1* in Familial Hypercholesterolemia

**DOI:** 10.1038/s41598-019-48402-y

**Published:** 2019-08-19

**Authors:** Magdalena Danyel, Claus-Eric Ott, Thomas Grenkowitz, Bastian Salewsky, Andrew A. Hicks, Christian Fuchsberger, Elisabeth Steinhagen-Thiessen, Thomas Bobbert, Ursula Kassner, Ilja Demuth

**Affiliations:** 1 Charité – Universitätsmedizin Berlin, corporate member of Freie Universität Berlin, Humboldt-Universität zu Berlin, and Berlin Institute of Health, Department of Endocrinology, Diabetes and Nutrition (including Lipid Metabolism), Berlin, Germany; 2 Institute of Medical Genetics and Human Genetics, Charité – Universitätsmedizin Berlin, corporate member of Freie Universität Berlin, Humboldt-Universität zu Berlin, and Berlin Institute of Health, Berlin, Germany; 3 Institute for Biomedicine, Eurac Research, Affiliated Institute of the University of Lübeck, Bolzano, Italy; 4 BCRT - Berlin Institute of Health Center for Regenerative Therapies, Charité University Medicine Berlin, Berlin, Germany

**Keywords:** Genetics research, Molecular medicine

## Abstract

Familial hypercholesterolemia (FH) is characterised by elevated serum levels of low-density lipoprotein cholesterol (LDL-C) and a substantial risk for cardiovascular disease. The autosomal-dominant FH is mostly caused by mutations in *LDLR* (low density lipoprotein receptor), *APOB* (apolipoprotein B), and *PCSK9* (proprotein convertase subtilisin/kexin). Recently, *STAP1* has been suggested as a fourth causative gene. We analyzed *STAP1* in 75 hypercholesterolemic patients from Berlin, Germany, who are negative for mutations in canonical FH genes. In 10 patients with negative family history, we additionally screened for disease causing variants in *LDLRAP1* (low density lipoprotein receptor adaptor protein 1), associated with autosomal-recessive hypercholesterolemia. We identified one *STAP1* variant predicted to be disease causing. To evaluate association of serum lipid levels and *STAP1* carrier status, we analyzed 20 individuals from a population based cohort, the Cooperative Health Research in South Tyrol (CHRIS) study, carrying rare *STAP1* variants. Out of the same cohort we randomly selected 100 non-carriers as control. In the Berlin FH cohort *STAP1* variants were rare. In the CHRIS cohort, we obtained no statistically significant differences between carriers and non-carriers of *STAP1* variants with respect to lipid traits. Until such an association has been verified in more individuals with genetic variants in *STAP1*, we cannot estimate whether *STAP1* generally is a causative gene for FH.

## Introduction

Autosomal-dominant hypercholesterolemia (FH, OMIM 143890) is one of the most common genetic disorders, characterised by severely elevated levels of low-density lipoprotein cholesterol (LDL-C). Its estimated prevalence ranges between 1:500 and 1:250^1–3^. Patients with FH are at substantial risk of developing atherosclerotic plaque deposition leading to premature coronary artery disease (CAD) or other cardiovascular disease (CVD)^4^. Diagnosis of FH is established clinically by pronounced hypercholesterolemia, xanthomas and corneal arcus, as well as history of premature CAD, other CVD, or other features suggestive of FH in the individual and first degree family members. On a molecular level, diagnosis of FH can be confirmed by presence of a heterozygous pathogenic mutation in one of three genes: *LDLR* (low density lipoprotein receptor, OMIM 606945)^5,6^, *APOB* (apolipoprotein B, OMIM 107730)^7,8^ and *PCSK9* (proprotein convertase subtilisin/ kexin, OMIM 607786)^9^. In some cases homozygosity, compound heterozygosity within the same gene, and double-heterozygotes for mutations in two of these genes can be observed^10^. Moreover, mutations in *LDLRAP1* (low density lipoprotein receptor adaptor protein, OMIM 605747) have been associated with familial hypercholesterolemia inherited in an autosomal-recessive manner (ARH, OMIM 603813)^11,12^.These patients can be treated by LDL-apheresis^13^ and PCSK9 inhibition with evolocumab in addition to statin and ezetimibe treatment^14,15^.

In FH patients, early diagnosis is essential for improvement of prognosis, reduction of cardiovascular mortality, and prevention of cardiovascular events by dietary and medical treatment. Clinically pre-diagnosed FH patients, e.g. based on the Dutch Lipid Clinic Network criteria (DLCNC)^16,17^, should undergo DNA testing which is an effective way to confirm diagnosis in an index patient and to cascade-screen families to identify other relatives with FH at risk for early CVD. Our working group has recently published data on the mutational spectrum in the genes *LDLR*, *APOB*, and *PCSK9* in 206 FH patients from Germany^18^. However, in our clinics, DNA testing of the three canonical FH genes was negative in approximately 60%, which is in the range reported by others^19^. Therefore, further research is necessary to identify new causative genes and to verify proposed candidate genes in independent cohorts to improve the molecular genetic diagnosis in FH patients who have not yet been confirmed by molecular genetic testing.

*STAP1* (signal transducing adaptor family member 1, OMIM 604298) encodes a docking protein also known as BRDG1 (BCR downstream signaling-1), which acts downstream of TEC (TEC protein tyrosine kinase) in B-cell antigen receptor signaling^20^. Using family-based linkage analysis in combination with whole exome sequencing in FH patients from the Netherlands, *STAP1* has been recently suggested to be the fourth FH gene^21^. However, the molecular mechanism by which STAP1 is supposed to act on cholesterol homeostasis remains unexplained.

This study represents a systematic molecular genetic analysis of *STAP1* in 75 unresolved FH patients, here defined as the Berlin FH cohort. In a separate, population-based cohort we evaluated association of carrier status for rare *STAP1* variants with total cholesterol (TC), low density lipoprotein cholesterol (LDL-C), high density lipoprotein cholesterol (HDL-C), and triglycerides (TG), since LDL-C levels have previously been postulated to be significantly higher in carriers of rare *STAP1* variants compared to wild type^21^.

## Materials and Methods

### The Berlin FH cohort

We included a total of 75 unrelated patients, who were diagnosed between 2012 and 2017 in the specialized Lipid Clinic at the Interdisciplinary Metabolism Centre, Charité - Universitätsmedizin Berlin, Germany, and who were initially screened negative for mutations in canonical FH genes (*LDLR*, *APOB*, *PCSK9*)^18^. Clinical diagnosis of FH was established as described previously^18^. In brief, we took the lipid parameters total cholesterol (TC), LDL-cholesterol (LDL-C), HDL-cholesterol (HDL-C), triglycerides (TG), and lipoprotein a [Lp(a)] as well as patients’ anamnesis, family history and physical examination into account. Additionally, we calculated a score according to the Dutch Lipid Clinic Network criteria (DLCNC)^16,17^, where a score >8 stands for “definite”, 6–8 for “probable”, 3–5 for “possible”, and <3 for “unlikely” diagnosis of FH. DLCN score calculation was not obligatory to enter genetic testing. LDL-C levels were compared to those obtained from 1600 older adults (age range 60–80 years) of Berlin Aging Study II (BASE-II)^19,22^ serving as the basic population and from patients with molecularly confirmed FH^18^. In patients receiving lipid lowering medication, we calculated medication-naïve LDL-C using conversion factors as described previously^18^.

### Mutation screening

Genomic DNA was extracted from peripheral blood by standard procedures. We performed Sanger sequencing of all 9 exons of STAP1 (NM_012108) including flanking intronic sequences. In 10 patients with negative family history we additionally analyzed the 9 exons and flanking intronic sequence of LDLRAP1 (NM_015627). Obtained sequences were analyzed using the Genious 9.1 software. Identified variants were checked using the database of the Exome Aggregation Consortium (ExAC, http://exac.broadinstitute.org/), the Human Gene Mutation Database (HGMD) and evaluated using PolyPhen^23^ and Mutation Taster^24^.

### Association of *STAP1* variants with lipid parameters in a population based cohort

To analyze the association of lipid parameters with carrier status of rare *STAP1* variants, we defined 20 participants of the Cooperative Health Research in South Tyrol (CHRIS) study carrying rare *STAP1* variants (ExAC minor allele frequency, MAF < 0.002) that were predicted to be disease-causing by MutationTaster as carriers. Further 100 participants of the CHRIS study were randomly selected as controls, i.e. non-carriers. Genetic analyses in this cohort were previously performed using Illumina HumanOmniExpressExome Bead Chip, which includes ~250,000 exonic variants^25^.

### Human subject recruitment, experimental procedures and research

Written informed consent was obtained from all participants. The Charité studies were approved by the ethics committee of the Charité – Universitätsmedizin Berlin, approval numbers EA2/089/14 and EA2/029/09; the CHRIS study was approved by the ethical committee of the Healthcare System of the Autonomous Province of Bolzano (Südtiroler Sanitätsbetrieb/Azienda Sanitaria dell’Alto Adige), protocol no. 21/2011. All experimental procedures and research was performed in accordance with relevant guidelines/regulations.

### Data analysis

Statistical analyses were performed using the IBM Statistical Package for the Social Sciences version 22.0 (IBM SPSS Statistics for Windows, Armonk, NY: IMB Corp. USA). Graphics were created using GraphPad Prism 6. Data from CHRIS study participants were additionally analyzed and plotted using R. *P*-values < 0.05 were considered to indicate statistical significance.

## Results

### Berlin FH cohort - Clinical characteristics

**Figure 1:**
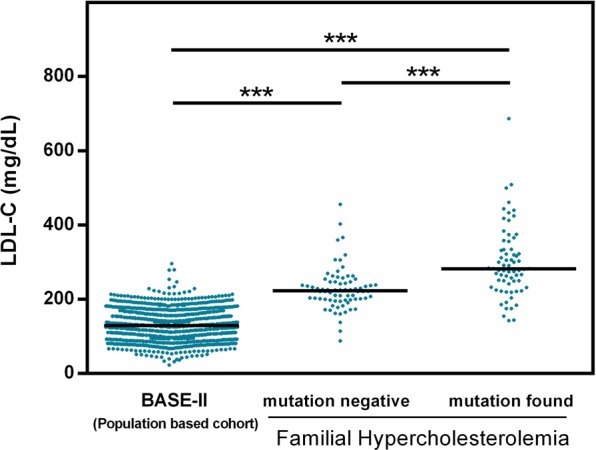
LDL-C serum level according to the mutation status of the genes *LDLR*, *APOB*, and *PCSK9*. LDL-C serum concentrations (mg/dl) are shown for a population based cohort (BASE-II, N = 1631)). LDL-C serum concentrations of the hypercholesterolemic patients with respect to the mutation status in one of the three FH genes *LDLR*, *APOB*, *PCSK9* (mutation negative (N = 75) vs. mutation found (N = 68)). Black lines indicate medians and dots single values. One-way ANOVA revealed significant differences between groups (*p* < 0.001). Post hoc Tukey’s test revealed significant differences between all possible pairs with ***indicating *p* < 0.001.

Characteristics and assessed lipid parameters are summarized in Table 1. Patients were admitted based on one of the following criteria or a combination of them: clinical signs of hyperlipidemia such as xanthomata and arcus lipoides, abnormality of lipid parameters, and positive family history of cardiovascular disease. Based on DLCN scores, diagnosis of FH was definite (>8) in five, probable (6-8) in 13, and possible (3–5) in 48 patients. In further nine, the score was <3. The latter patients were still included in the current study since moderately elevated lipid levels have been reported in association with *STAP1* variants^21^. 64% were female and 36% were male. Except for HDL-C, lipid parameters did not significantly differ between males and females (Supplementary Fig. S1). The median age was 55 years. TC and LDL-C levels tended to be higher at higher age (Supplementary Fig. S2). As shown in Fig. 1, LDL-C levels of the Berlin FH cohort were significantly higher when compared to data from a population based BASE-II cohort. However, the levels were significantly lower when compared to confirmed FH patients with known mutations in *LDLR* and *APOB* (each *p* < 0.001).

**Table 1:** Characteristics of the Berlin FH cohort.

Variable	Berlin FH cohort (*n* = 75)	
Sex		
Female (%)	64	
Male (%)	36	
Age (years)		
Mean ± SD	53 ± 13	
Range	25–81	
TC in mg/dl		
Median (IQR)	313 (272–344)	
Range	190–677	
LDL-C in mg/dl		
Median (IQR)	223 (199–247)	
Range	88–456	
HDL-C in mg/dl		
Median (IQR)	58 (51–69)	
Range	30–117	
TG in mg/dl		
Median (IQR)	175 (125–261)	
Range	45–1127	
DLCN-Score	Interpretation	Number of individuals
<3	Unlikely FH	09
3–5	Possible FH	48
6–8	Probable FH	13
>8	Definite FH	05

The table displays sex, age, and lipid parameters (TC, total cholesterol; LDL, low density lipoprotein; HDL, high density lipoprotein; TG, triglycerides) as well as calculated DLCN (Dutch Lipid Clinic Network criteria) score and its distribution. IQR, interquartile Range.

### Berlin FH cohort - Sequencing results

In all 75 patients sequencing analysis revealed no deleterious mutation in *STAP1*, i.e. nonsense or frameshift-mutations. All three identified variants within the coding sequence are listed in Supplementary Table S1. Despite two variants predicted to be benign, we identified one individual carrying a rare single nucleotide variant (SNV), *rs199787258*, with an estimated MAF of 0.0003 (ExAC). This *STAP1* variant, c.526 C > T, p.(Pro176Ser) was described as disease-causing mutation in the Online Table III by Fourier and colleagues^21^ and recently by Blanco-Vaca and coworkers^26^. In the Berlin FH cohort, the 78-year-old male patient carrying this variant presented with abnormal lipid parameters: elevated TC of 450 mg/dl (reference <200 mg/dl), elevated LDL-C of 360 mg/dl (reference <130 mg/dl), HDL-C of 68 mg/dl (reference >55 mg/dl), elevated TG of 222 mg/dl (reference <200 mg/dl), and Lp(a) of 190 mg/l (reference <300 mg/l). Segregation within the family revealed that his 49-years old son carried the same variant with following lipid parameters: elevated TC of 270 mg/dl, elevated LDL-C of 159 mg/dl, reduced HDL-C of 26 mg/dl, and elevated TG of 498 mg/dl. Unfortunately, no other relatives were available for the segregation study. Sequencing of *LDLRAP1* coding sequence in the 10 patients with negative family history to exclude autosomal-recessive hypercholesterolemia (ARH) revealed no pathogenic variant.

### CHRIS cohort - lipid parameters in carriers of rare *STAP1* variants

To gain further insights into the association of rare *STAP1* variants with abnormal lipid parameters, we therefore tested for association in an independent, population-based cohort, the Cooperative Health Research in South Tyrol (CHRIS) study^25^. Characteristics of the *STAP1* variants identified in 20 participants, here defined as ‘carriers’ of the CHRIS cohort, are summarized in Table 2. Of note, one of them carried the same *STAP1* variant (*rs199787258*, c.526 C > T, p.Pro176Ser) that we had observed in the Berlin FH cohort, suddenly associated with almost normal lipid parameters at the age of 54 years: TC 206 mg/dl (reference TC <200 mg/dl), LDL-C 111 mg/dl (reference LDL-C <115 mg/dl), HDL-C 82 mg/dl (reference HDL-C in females >45 mg/dl), and TG 81 mg/dl (reference TG 30–150 mg/dl).

**Table 2:** Rare *STAP1* variants observed in the CHRIS cohort.

*STAP1* variant	Location (hg19)	A1	A2	MAF	DNA changes	AA changes	Mutation taster	PolyPhen	Number of carriers (total *n* = 20)
rs146545610	4:68449380	G	A	0.0001	c.619 G > A	p.Asp207Asn	disease causing	benign	3
rs141647540	4:68424562	G	A	0.0002	c.35 G > A	p.Arg12His	disease causing	probably damaging	14
rs149803575	4:68447073	G	C	0.0017	c.414 G > C	No changes	disease causing	benign	2
rs199787258	4:68447185	C	T	0.0003	c.526 C > T	p.Pro176Ser	disease causing	probably damaging	1

Given are the dbSNP IDs, chromosomal coordinates according to human genome GRCh37 (hg19), the effective allele A1, the alternative allele A2 on the forward strand, the minor allele frequency (MAF), the change on DNA level where position 1 of the “c” coordinate is the A of the ATG start (NM_012108). AA changes describes the estimated change on amino acid level, and the columns *MutationTaster* and *PolyPhen* give the prediction of the corresponding tools. The last column indicates the number of individuals carrying the distinct variant. Note, that the *STAP1*-variant rs199787258 was also identified in two individuals of the Berlin FH cohort.

For further analyses, we randomly selected 100 CHRIS study participants in whom rare *STAP1* variants, i.e. MAF <0.002, were excluded, to characterize the distribution of the lipid parameters and termed them ‘non-carriers’.

**Figure 2:**
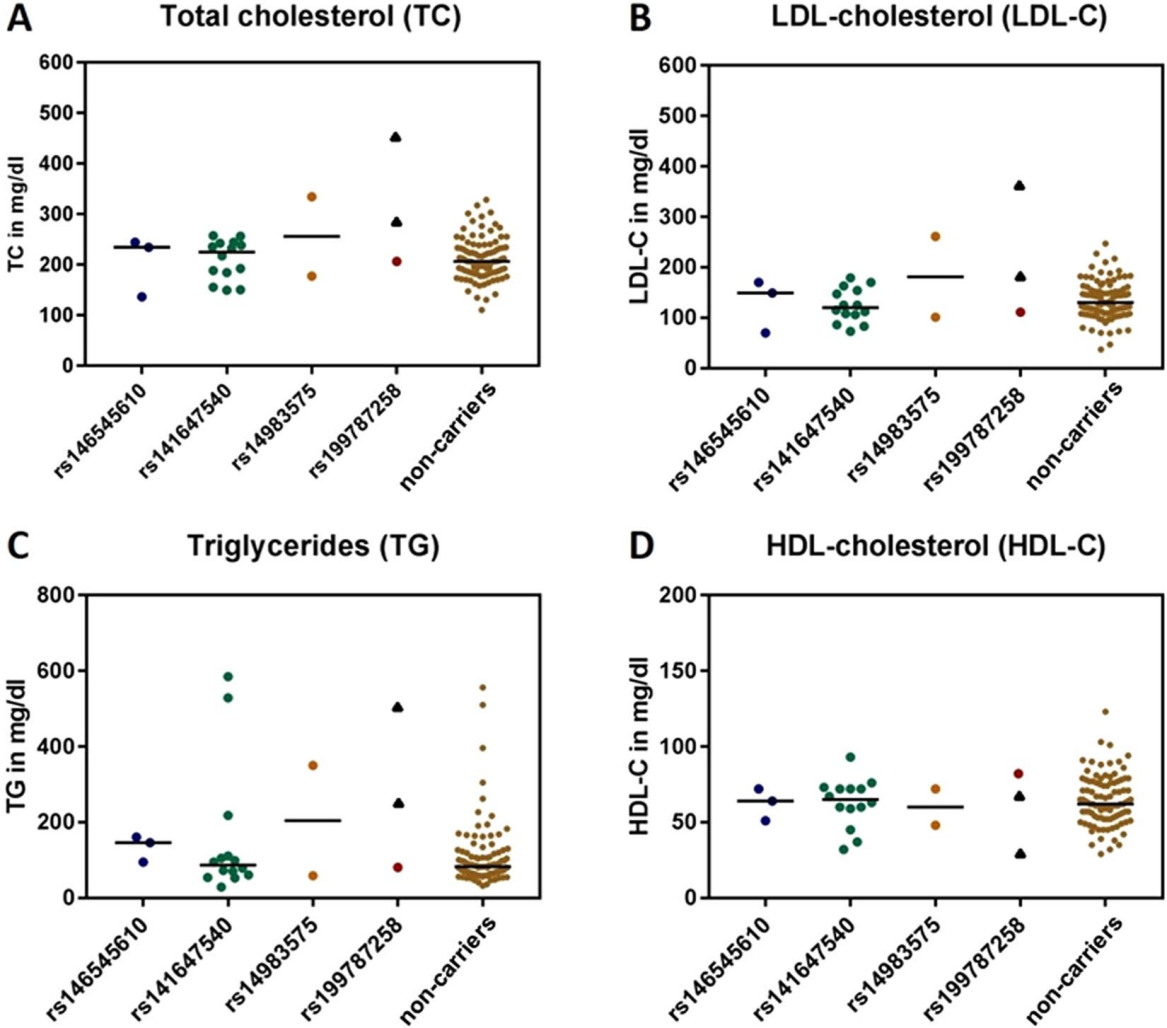
Lipid values according to *STAP1* variant carrier status. For carriers of rare *STAP1* variants [rs14655610 (N = 3), rs141647540 (N = 14), rs14983575 (N = 2), and rs199787258 (N = 1)], as well as *STAP1* non-carriers (N = 100) individual values of (**A**) Total cholesterol (TC), (**B**) LDL-cholesterol (LDL-C), (**C**) Triglycerides (TG), and (**D**) HDL cholesterol (HDL-C) are depicted. Colored dots indicate values of participants of the CHRIS study, i.e. 20 carriers and 100 non-carriers. Bars indicate median values. To allow for comparison, black triangles indicate values of the two individuals of the Berlin FH cohort. Since these individuals originate from a different cohort and were assessed in a different laboratory, calculation of a median is not applicable for rs199787258.

Visualization of the lipid parameters (Fig. 2) revealed that lipid values TC and LDL-C of the individual from the Berlin FH cohort were higher than values of the individuals of the CHRIS cohort, no matter whether they were carriers or non-carriers. The values of the son of the index patient of the Berlin FH cohort carrying the same variant were within the distribution of non-carriers of the CHRIS cohort. In contrast to LDL-C, one of the key parameters used to establish the diagnosis of FH, we observed TG levels comparable to the Berlin FH index patient both in some carriers and some non-carriers.

We also tested the hypothesis that carriers of a rare variant in *STAP1* have higher lipid parameters using an unpaired two-sided Mann-Whitney U test. However, no statistically significant differences were observed (Supplementary Table S2, Supplementary Fig. S3). Additionally, we raised the question whether abnormality of lipid levels taken as a categorical variable might be more frequent in carriers versus non-carriers. Although we observed a marginal preponderance of elevated TC levels, a slight preponderance of normal (!) LDL-C levels, a slight preponderance of elevated TG levels, and a slight preponderance of reduced HDL-C levels in carriers, none of these differences revealed statistical significance (Supplementary Table S3, Supplementary Fig. S4).

Finally, we excluded that confounders such as sex or age might have blurred association. Of the CHRIS study participants included into these analyses, 67 were females and 53 were males. Sex ratio in *STAP1* variant carriers was nine males to 11 females, i.e. 45% versus 55%, and in non-carriers 44 males to 56 females, i.e. 44% versus 56%. Except for HDL-C, lipid parameters did not significantly differ between males and females (Supplementary Fig. S5). Median age was 48 years in all participants, 55 years in carriers, and 47 years in non-carriers. Higher TC as well as higher LDL-C were associated with higher age (each *p* < 0.001), and TG slightly increased with increasing age (*p* < 0.05) (Supplementary Fig. S6). In summary, sex distribution can be excluded as a confounder, and the age structure would lead to false positive but not false negative results, if at all. Additionally, linear regression analyses with lipid parameters as dependent variables and adjusting for sex, age and carrier status (yes/no) revealed no significant differences in lipid levels between carriers and non-carriers (data not shown).

## Discussion

Based on family studies and next-generation sequencing (NGS), three genes in addition to the established FH genes were identified, in which mutations may be causing significantly elevated LDL-C levels and possibly the clinical phenotype of FH: *STAP1* (signal transducing adaptor protein family 1), *LIPA* (lysosomal acid lipase) and *PNPLA5* (patatin-like phospholipase-domain-containing family)^21,27,28^. To our knowledge, confirmation of the genes *STAP1* and *PNPLA5* as well as variants within them to be causative for FH in independent studies is still pending.

Since allocation to the Berlin FH cohort is based on abnormal lipid parameters, association of a genetic variant with elevated lipid parameters in this cohort could be biased. Further, segregation of the identified *STAP1* variant c.526 C > T, p.(Pro176Ser) in two first degree relatives can be observed by chance in 50%, and segregation within families can also be biased since individuals from the same families do not only share genetic traits but may additionally share environment, culture, and habits. The segregation study of this variant within the family described by Blanco-Vaca and coworkers also indicates a polygenic contribution to hypercholesterolemia. There, the daughter of the index patient, who also carried the c.526 C > T, p.(Pro176Ser) variant, had no hypercholesterolemia, in contrast to her brother, who as a non-carrier showed the phenotype^26^. Taken together, both previous findings and findings in our cohort cannot clarify whether the identified *STAP1* variant is incidental or causative for the observed phenotype.

Genome wide association studies such as the one by Teslovich *et al*.^29^ suggested 95 loci for blood lipids. However, they obtained no hit for *STAP1* alias *BRDG1*. The linkage interval on chromosome 4p15.1-q13.3 (hg19; chr4:27,700,001-76,300,000) obtained by Fouchier *et al*. encompasses 48.6 Mb^21^. Comparison of both studies revealed that the genes *KLHL8* and *SLC39A8* are the only candidate genes having an impact on lipid traits localized on chromosome 4. However, both are localized outside the linkage interval. Thus, we cannot exclude that there might be yet another candidate gene for FH hidden within this linkage interval which might also segregate with the observed phenotype.

Paquette and colleagues used genetic risk scores (GRSs) to evaluate the polygenetic modification of FH phenotype^30^. This approach included 13 common SNPs on chromosome 4 of which two, *rs17087335* in *NOA1*, and *rs10857147* (between *PRDM8* and *FGF5*) are localized within the linkage interval described by Fouchier and colleagues. However, there was no informative common SNP in *STAP1*.

Pirillo *et al*. used exome screening in an Italian cohort of FH patients where they confirmed diagnosis in 67% by molecular genetic analysis. They used a DLCN score >5 as inclusion criterion^31^. Since Fouchier and colleagues suggested that mutations in *STAP1* are associated with less severe elevation of lipid parameters, we did not use such a stringent DLCN score cut-off in our study. Thus, the expected mutation detection rate should be lower in comparison to the one reported by Pirillo and colleagues^31^.

If one takes all patients with hypercholesterolemia or lipid lowering medication into account, one would expect to detect causative mutations in one of the known FH genes in 2.1% and 2.2% of cases, respectively^32^.

The frequency of rare *STAP1* variants predicted to be pathogenic or possibly pathogenic in the FH4 cohort of Fouchier *et al*. was 1.3% (5 of 400 individuals). We identified one carrier in 75 unrelated individuals (1/75 = 1.3%)^21^. Thus, our study on the Berlin FH cohort confirms that *STAP1* variants are fairly rare.

It might be possible that a substantial number of the unresolved cases in the Berlin FH cohort carry a single mutation in one of the known or unknown FH genes that cannot be detected by conventional methods. It is a limitation of our study, that we have not evaluated polygenic FH variants such as the *APOE* rs429358 systematically or screened for mutations in this gene, as these were shown to be associated with hypercholesterolemia^33–35^. The use of a less stringent DLCN cut-off might also lead to inclusion of a significant fraction of patients with polygenic hypercholesterolemia (PHC). Here, affected individuals carry a greater-than-average number of common cholesterol-raising genetic variants that collectively have a detectable effect on LDL-C levels^36^. Additionally, one might even speculate that epigenetic DNA modifications caused by *in utero* exposure to hypercholesterolemia might influence LDL-C levels and possibly the clinical phenotype of FH. Indeed, there are differentially methylated regions that are associated with serum LDL cholesterol, and DNA methylation signatures link prenatal malnutrition to growth and adverse metabolic phenotype in the offspring^37^. We note, that 10/75 individuals, i.e. 13% of the (unresolved) Berlin FH cohort were born between 1936 and 1950.

The participants of the CHRIS study were assigned to carrier and control groups based on the presence or absence of one of the rare variants in *STAP1* (MAF <0.002). Thus, we cannot exclude presence of other rare variants in *STAP1* in members of the control group such as deep intronic or enhancer variants that cannot be captured by the genotyping strategy used in the CHRIS study.

Our statistical analysis contains the uncertainty that lipid lowering medication might have blurred association between *STAP1* variants and lipid parameters. However, only six of the 120 selected participants have stated that they take lipid lowering medication. Thus, we assume, that this will probably have no substantial effect on our result.

Another limitation of our study is the limited number of carriers of identical *STAP1* variants. Since statistical significance is dependent on sample size, we pooled four different rare SNPs in *STAP1* to obtain a sample size of at least 5. In contrast, Fouchier and colleagues performed segregation of large numbers of carriers versus non-carriers within families resulting in rather homogeneous subgroups. Thus, distinct variants within these families are associated with abnormal lipid parameters and the large sample size can give significance even for slight differences.

Genetic sequencing analyses are commonly used not only to confirm the diagnosis, but also to identify other family members at risk of cardiovascular disease. The elevation of lipid parameters associated with pathogenic variants in *STAP1* is rather mild in comparison to pathogenic variants in the canonical FH genes *LDLR*, *APOB*, and *PCSK9*. In addition, our study revealed that at least in the Berlin FH cohort, rare sequence variants that might be pathogenic and deleterious mutations are not common. Thus, we conclude that the positive predictive value of *STAP1* analysis will be comparably small. Based on our data, we can therefore not postulate that *STAP1* analysis has necessarily to be included into molecular assessment of cardiovascular risk by sequencing panels. However, further knowledge about *STAP1* sequence variants in FH patients could help to estimate whether specific domains in this gene might be associated with a higher risk to develop FH, or whether rare genetic variants in *STAP1* may modify the disease phenotype of FH. Further work, including *in vitro* functional studies, should focus on the molecular interactions of *STAP1* to verify the role in pathogenesis of FH.

The next step will be retrospective analysis of *APOE*^33^, *LIPA* and *PNPLA5*^27^ in the Berlin FH cohort, since mutations in these genes may cause significantly elevated LDL-C levels and possibly the clinical phenotype of FH. Additionally, determination of the 6 SNPs score described by Futema^34^ and colleagues in the Berlin cohort would be helpful to further delineate the genetic bases of LDL-C levels in this cohort. In prospective studies on FH patients, exome sequencing combined with more stringent inclusion criteria might be reasonable.

## Supplementary information

Supplementary information

## Data Availability

Due to concerns for participant privacy, data are available only upon request. External scientists may apply to the internal committee of the study of interest (CHRIS or BASE-II) for data access.
