# Supplementary material for: Evaluation of the role of STAP1 in Familial Hypercholesterolemia
Source: Sci Rep. 2019 Aug 19;9:11995. doi: 10.1038/s41598-019-48402-y (PMC6700100; doi:10.1038/s41598-019-48402-y)
Supplement: Supplementary file 1 — Supplementary information [file 41598_2019_48402_MOESM1_ESM.pdf]

Evaluation of the role of *STAP1* in Familial Hypercholesterolemia

Magdalena Danyel[1,2], Claus-Eric Ott[2], Thomas Grenkowitz[1], Bastian Salewsky[1], Andrew Hicks[3], Christian Fuchsberger[3], Elisabeth Steinhagen-Thiessen[1], Thomas Bobbert[1],Ursula Kassner[1], Ilja Demuth[1,4]

[1] Charité – Universitätsmedizin Berlin, corporate member of Freie Universität Berlin, Humboldt-Universität zu Berlin, and Berlin Institute of Health, Lipid Clinic at the Interdisciplinary Metabolism Center, Germany

[2] Institute of Medical Genetics and Human Genetics, Charité – Universitätsmedizin Berlin, corporate member of Freie Universität Berlin, Humboldt-Universität zu Berlin, and Berlin Institute of Health, Berlin, Germany

[3] Center for Biomedicine, European Academy of Bozen/Bolzano (EURAC), affiliated to the University of Lübeck, Via Galvani 31, Bolzano 39100, Italy

[4] BCRT - Berlin Institute of Health Center for Regenerative Therapies, Charité University Medicine Berlin

Corresponding author:
Ilja Demuth (PhD)
Charité - Universitätsmedizin Berlin
Lipid Clinic at the Interdisciplinary Metabolism Center,
Biology of Aging Group
Augustenburger Platz 1
13353 Berlin
Email: ilja.demuth@charite.de
Phone: +49 30 450 569 143
Fax: +49 30 450 566 904

Contents

**Supplementary Table S1**	Berlin FH cohort. Identified variants in *STAP1*.
**Supplementary Fig. S1**	Berlin FH cohort. Lipid parameters vs. sex
**Supplementary Fig. S2**	Berlin FH cohort. Lipid parameters vs. age
**Supplementary Fig. S3**	CHRIS cohort. Lipid parameters vs. carrier status
**Supplementary Table S2**	Lipid parameters vs. *STAP1* rare variant carrier status in the CHRIS cohort
**Supplementary Table S3**	Frequency of abnormal lipid parameters in *STAP1* variant carriers vs. non-carriers
**Supplementary Fig. S4**	CHRIS cohort. Abnormality of lipid parameters vs. carrier status.
**Supplementary Fig. S5**	CHRIS cohort. Lipid parameters vs. sex
**Supplementary Fig. S6**	CHRIS cohort. Lipid parameters vs. age

STAP1 variant	Location (hg19)	A1	A2	MAF	DNA changes	AA changes	Mutation taster	PolyPhen
rs11556614	4:68442968	A	G	0.5180	c.354A>G	p.Thr118Thr	polymorphism	benign
rs11556615	4:68447040	C	T	0.1923	c.381C>T	p.Asn127Asn	polymorphism	benign
rs199787258	4:68447185	C	T	0.0003	c.526C>T	p.Pro176Ser	disease causing	Probably damaging

Supplementary Table 1. Berlin FH cohort. Identified variants in *STAP1*. Listed are variants within the coding sequence of *STAP1*. Given are the dbSNP IDs, chromosomal coordinates according to human genome GRCh37 (hg19), the effective allele A1, the alternative allele A2 on the forward strand, the minor allele frequency (MAF), the change on DNA level where position 1 of the "c" coordinate is the A of the ATG start (NM_012108). AA changes describes the estimated change on amino acid level, and the columns MutationTaster and PolyPhen give the prediction of the corresponding tools.

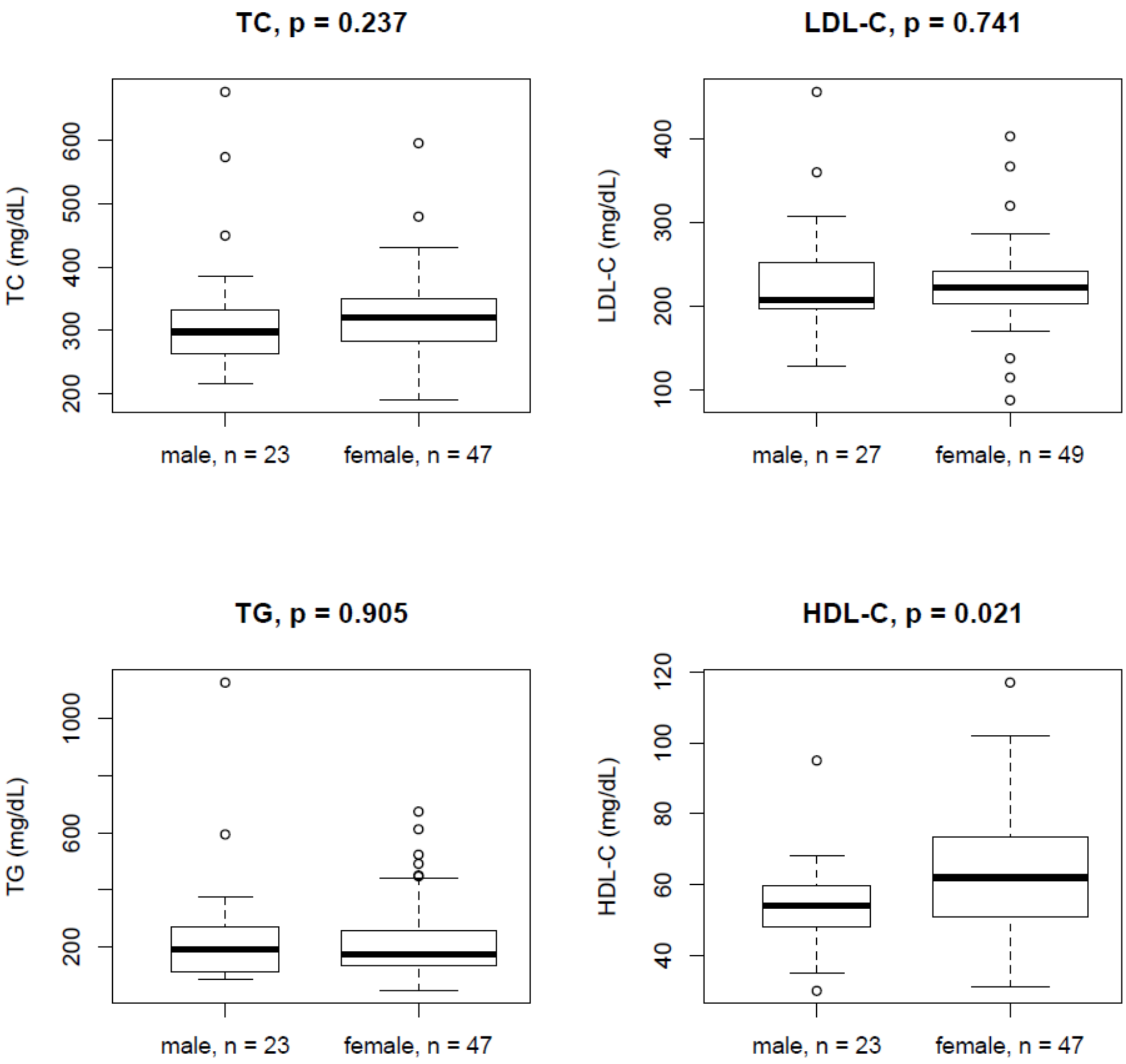

Supplementary Figure S1: Berlin FH cohort. Lipid parameters versus sex. Total cholesterol (TC), low-density lipoprotein cholesterol (LDL-C), and triglycerides (TG) were not significantly different in males versus females of the Berlin cohort of total of 76 FH patients, who were tested negative for mutations in *LDLR*, *APOB*, and *PCSK9*. In contrast, high-density lipoprotein cholesterol was significantly higher in females (p <0.05). Association of factor variable and metric parameters was estimated using unpaired two-sided Mann-Whitney U test.

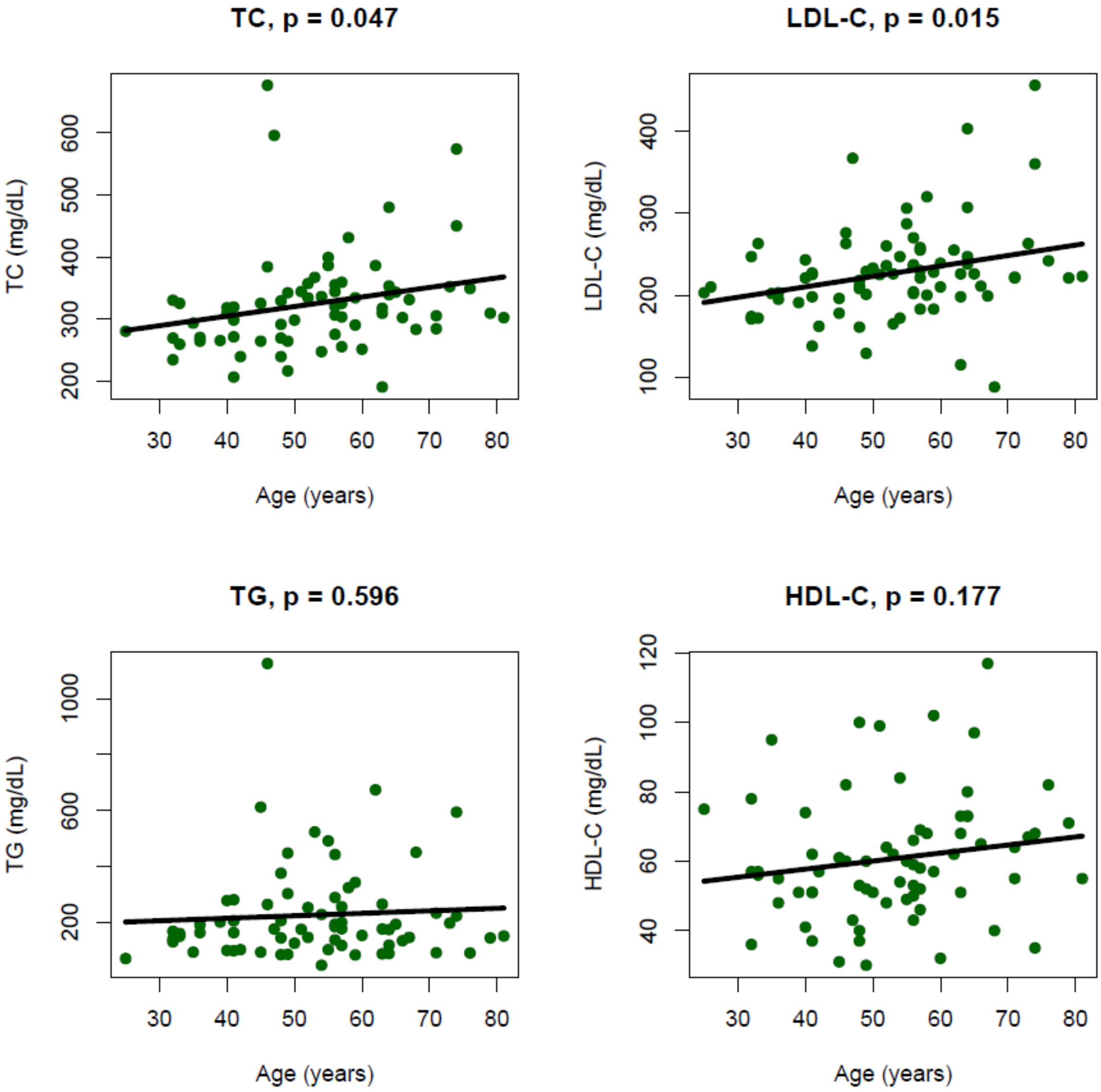

Supplementary Figure S2: Berlin FH cohort. Lipid parameters versus age. Total cholesterol (TC) and low-density lipoprotein cholesterol (LDL-C) are significantly and positively associated with age (slope 1.542 (TC), 1.273 (LDL-C); each p <0.05). However, with increasing age variance of LDL-C increases, too. In contrast, there was no significant association of triglycerides (TG) and high-density lipoprotein cholesterol (HDL-C) with age. Association between two metric variables was estimated using nonparametric two-sided Mann-Whitney U test. Dots indicate individual values. Linear regression lines are plotted in black.

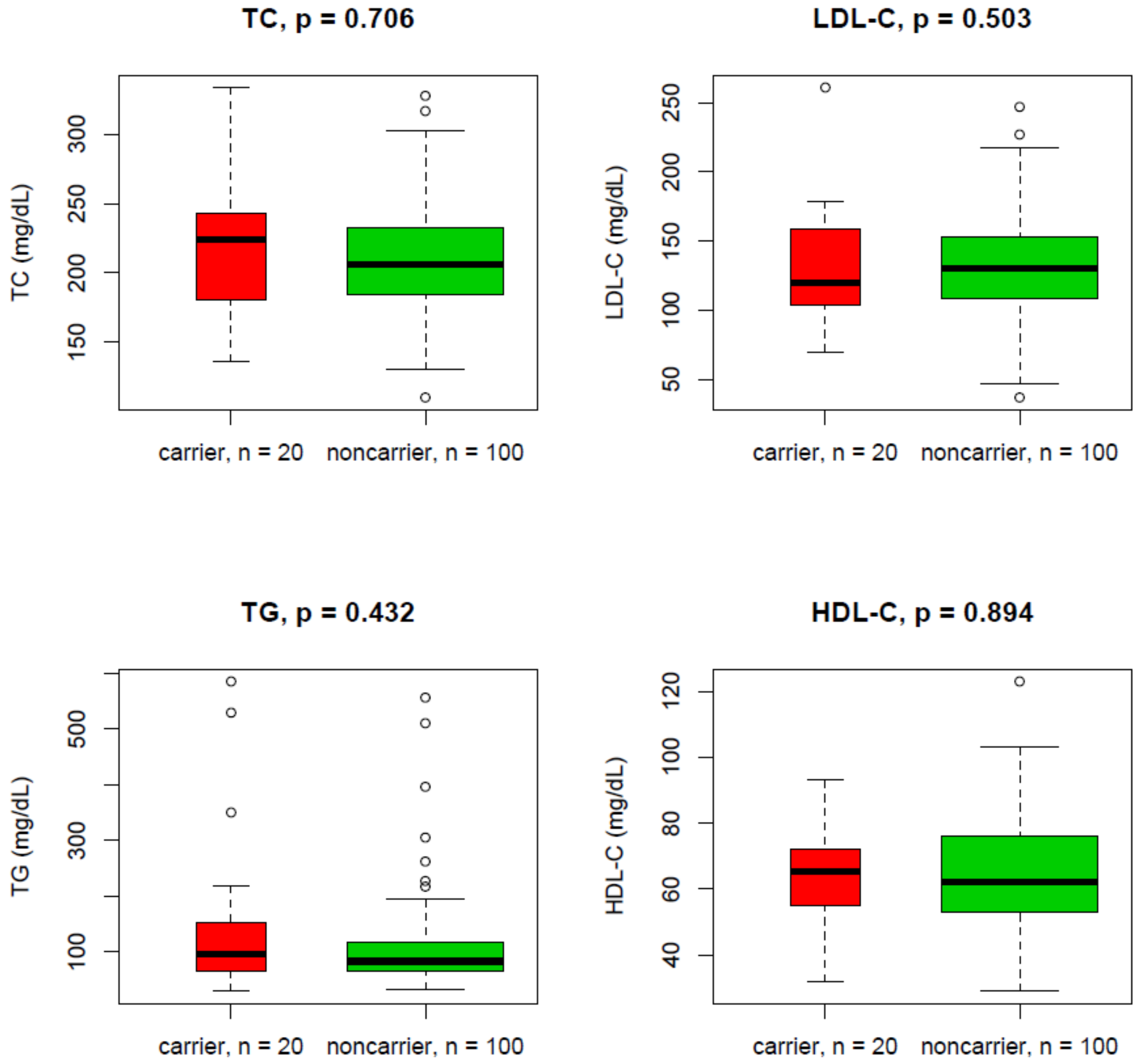

Supplementary Figure S3: CHRIS cohort. Lipid parameters versus carrier status. We found no statistical association of carrier status of rare variants in *STAP1* with lipid parameters total cholesterol (TC), low-density lipoprotein cholesterol (LDL-C), triglycerides (TG), and high-density lipoprotein cholesterol (HDL-C). Association of factor variable and metric parameters was estimated using unpaired two-sided Mann-Whitney U test. Carriers are depicted in red, non-carriers in green.

Supplementary Table S2

Parameter	Carriers (*n*=20)	Non-carriers (*n*=100)	*p*-value (Mann-Whitney U test)
**TC in mg/dl**			*p* =0.706
Median (IQR)<br>Range	225 (182-243)<br>136-334	206 (185-233)<br>110-328	
**LDL-C in mg/dl**			*p* =0.503
Median (IQR)<br>Range	120 (105-156)<br>70-261	130 (109-153)<br>37-247	
**TG in mg/dl**			*p* =0.432
Median (IQR)<br>Range	83 (69-150)<br>29-585	83 (65-118)<br>32-556	
**HDL-C in mg/dl**			*p* =0.893
Median (IQR)<br>Range	66 (57-72)<br>32-93	62 (53-76)<br>29-123	

Supplementary Table S2. Lipid parameters vs. *STAP1* rare variant carrier status in the CHRIS cohort. A nonparametric two-sided Mann-Whitney U test was performed to estimate significance. Corresponding plots are shown in Supplementary Fig. S3.

Supplementary Table S3

Parameter	Normal in carriers	Abnormal in carriers	Normal in non-carriers	Abnormal in non-carriers	Chi-squared *p*-value
TC	8	12	43	57	*p*=0.804
LDL-C	10	10	33	67	*p*=0.148
TG	15	5	84	16	*p*=0.334
HDL-C	18	2	94	6	NA

Supplementary Table S3. Frequency of abnormal lipid parameters in *STAP1* variant carriers vs. non-carriers. For analysis of the CHRIS dataset we used the following reference values: TC <200 mg/dl, LDL-C <115 mg/dl, TG 30-150 mg/dl, HDL-C in females >45 mg/dl, in males >40 mg/dl. Shown are lipid parameters and numbers of individuals. Chi-square test was performed to estimate significance. Since the total number of carriers with reduced HDL-C levels is <5, calculation of chi-square test should not be performed. Corresponding mosaic plots are shown in Supplementary Fig. S4.

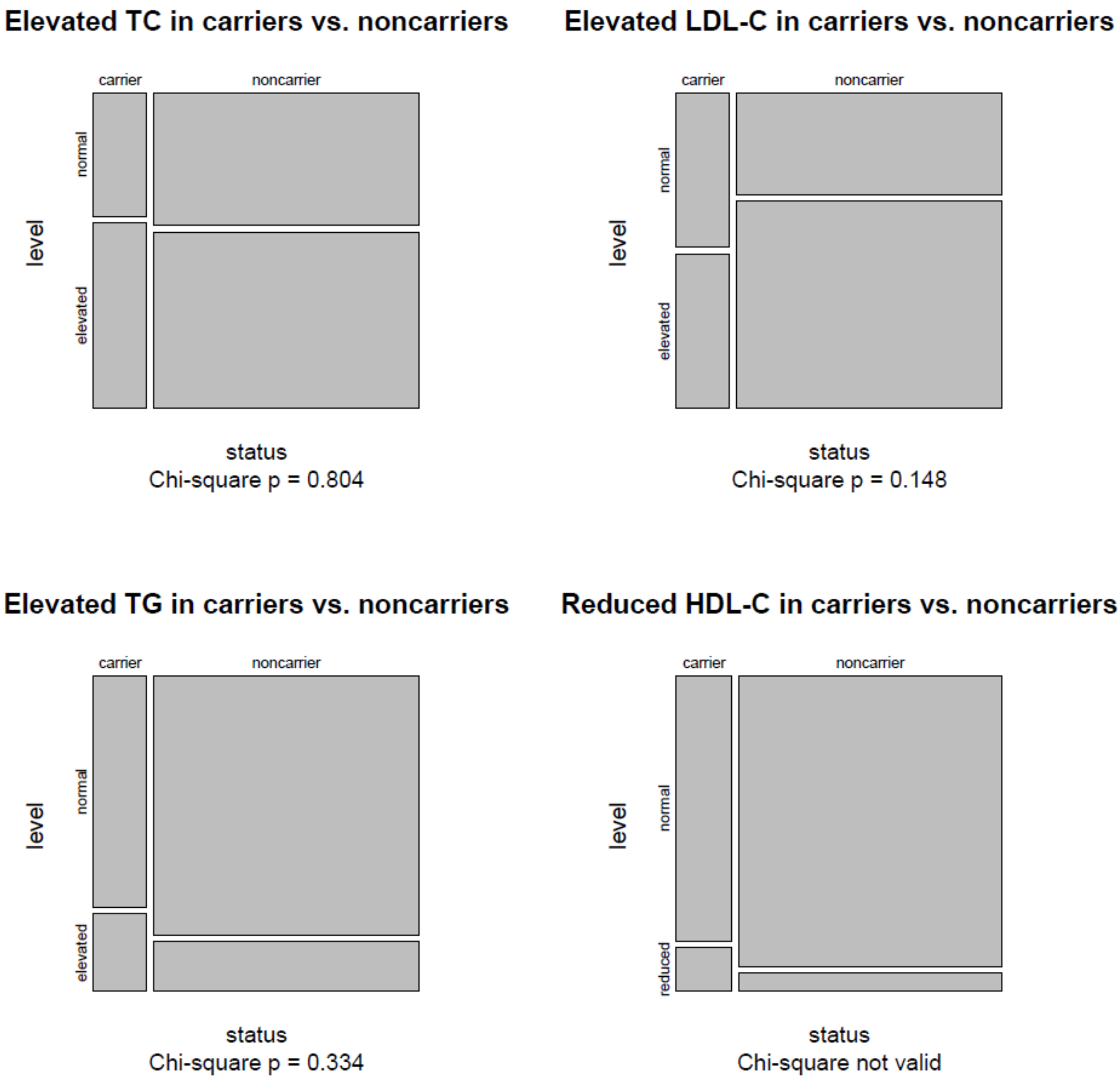

Supplementary Figure S4: CHRIS cohort. Abnormality of lipid parameters versus carrier status. Chi-square tests revealed no statistically significant preponderance of lipid parameter abnormalities in carriers of rare *STAP1* variants. The data are visualized using mosaic diagrams to examine the relationship among categorical variables carrier vs. non-carrier and lipid parameter above or below the reference threshold.

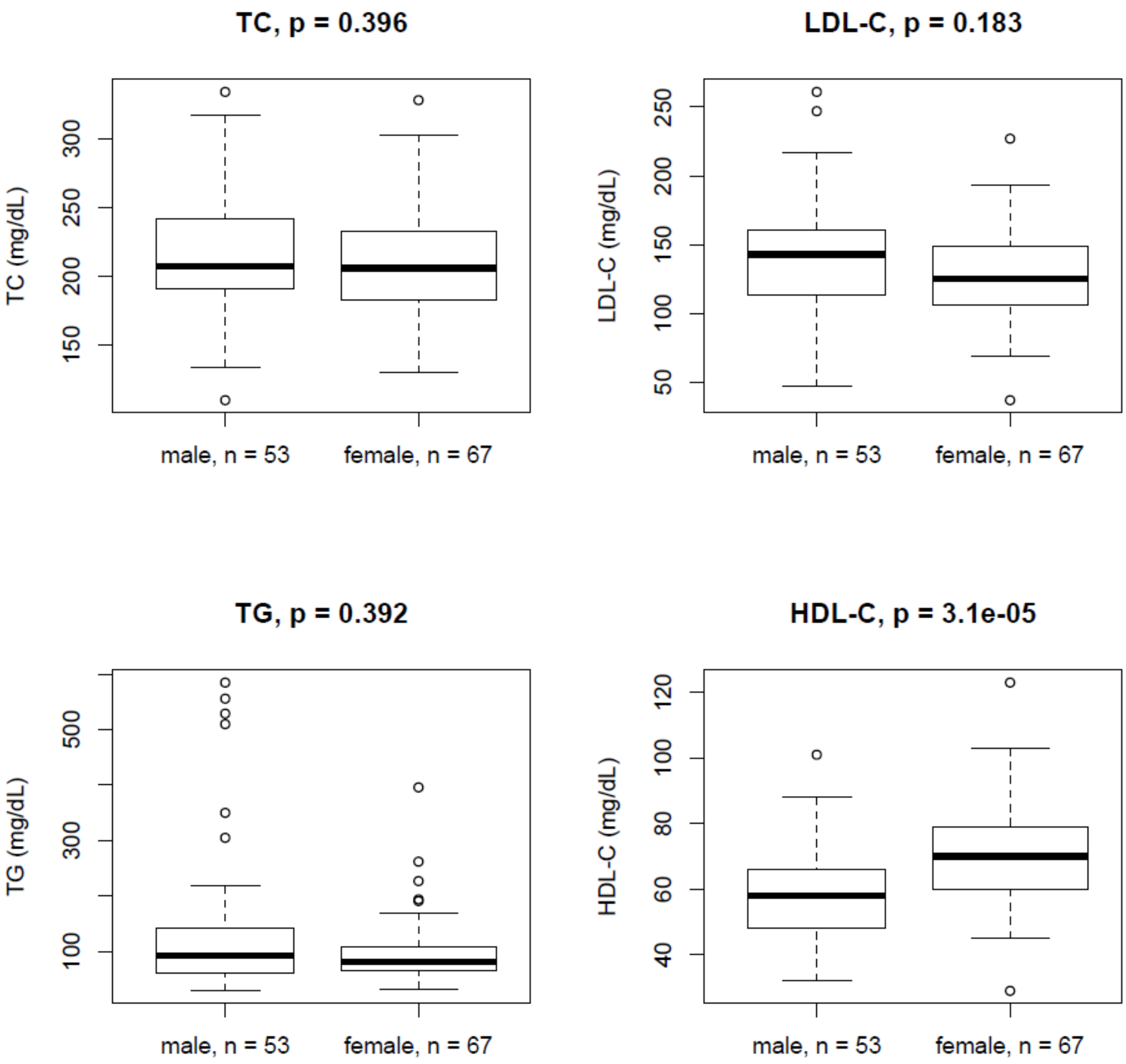

Supplementary Figure S5: CHRIS cohort. Lipid parameters versus sex. Total cholesterol (TC), low-density lipoprotein cholesterol (LDL-C), and triglycerides (TG) are not significantly different in males versus females of the CHRIS cohort of total of 120 participants. In contrast, high-density lipoprotein cholesterol is significantly higher in females (p <0.0001). Association of factor variable and metric parameters was estimated using unpaired two-sided Mann-Whitney U test.

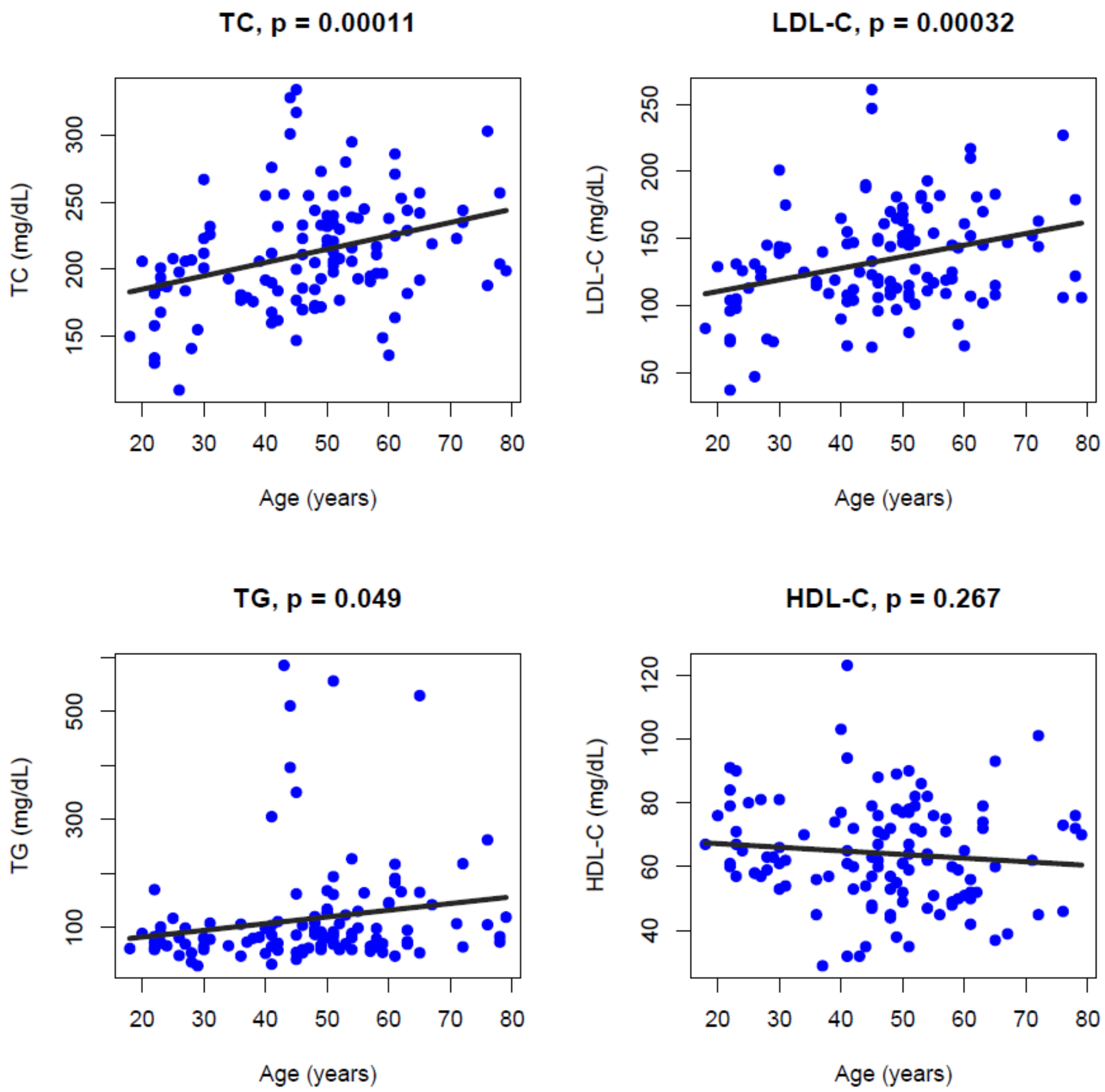

Supplementary Figure S6: CHRIS cohort. Lipid parameters versus age. Increasing total cholesterol (TC), low-density lipoprotein cholesterol (LDL-C), and triglycerides (TG) are significantly associated with increasing age (slope 0.995 (TC), slope 0.864 (LDL-C), slope 1.242 (TG); each p <0.05). In contrast, there was no significant association of high-density lipoprotein cholesterol (HDL-C) with age. Association between two metric variables was estimated using nonparametric two-sided Mann-Whitney U test. Dots indicate individual values. Regression lines are plotted in black.
